# Screening for onconeural antibodies in neuromyelitis optica spectrum disorders

**DOI:** 10.1186/s12883-016-0779-9

**Published:** 2017-01-10

**Authors:** Benjamin Berger, Tilman Hottenrott, Sebastian Rauer, Oliver Stich

**Affiliations:** Department of Neurology and Neurophysiology, Medical Center—University of Freiburg, Faculty of Medicine, Breisacher Strasse 64, D-79106 Freiburg, Germany

**Keywords:** Aquaporin-4, Neuromyelitis optica spectrum disorders, NMOSD, Onconeural antibodies, Paraneoplastic

## Abstract

**Background:**

Some so-called “non-classical” paraneoplastic neurological syndromes (PNS), namely optic neuritis and myelitis, clinically overlap with neuromyelitis optica spectrum disorders (NMOSD), and conversely, in cancer-associated NMOSD, a paraneoplastic etiology has been suggested in rare cases. Therefore, we retrospectively investigated the prevalence of onconeural antibodies, which are highly predictive for a paraneoplastic etiology, and the prevalence of malignancies in NMOSD patients.

**Methods:**

We retrospectively screened 23 consecutive patients from our clinic with NMOSD (13 were anti-aquaporin-4 [AQP4] antibody positive, 10 were AQP4 negative) for onconeural antibodies using an immunoblot.

**Results:**

All patients were negative for a broad spectrum of antibodies targeting intracellular onconeural antigens (Hu, Yo, Ri, CV2/CRMP5, Ma1, Ma2, Zic4, SOX1, Tr, and amphiphysin). Notably, only two patients had a malignancy. However, neoplastic entities (astrocytic brain tumor and acute myeloid leukemia) were not typical for PNS.

**Conclusions:**

Our data suggest that there is no need to routinely screen anti-AQP4 antibody positive NMOSD patients with a typical presentation for onconeural antibodies. Furthermore, absence of these antibodies in NMOSD, which is typically non-paraneoplastic, confirms their high specificity for PNS.

## Background

Neuromyelitis optica (NMO) is a rare, immune-mediated, demyelinating disorder of the central nervous system (CNS), typically presenting with relapsing optic neuritis (ON) and/or ≥ three vertebral segment longitudinally extensive transverse myelitis (LETM) [1, 2]. Pathogenetic antibodies targeting the water channel protein aquaporin-4 (AQP4) are found in the majority of patients with NMO [3]. Since their discovery, the spectrum of clinical manifestations within the CNS associated with AQP4 antibodies has expanded [4]. Therefore, diagnostic criteria have recently been revised, introducing the term “neuromyelitis optica spectrum disorders (NMOSD)” [5]. According to these revised criteria, an NMOSD diagnosis can also be established in absence of anti-AQP4 antibodies. For simplicity, in the following, the term “NMOSD” is consistently used for both NMO and NMOSD.

Paraneoplastic neurological syndromes (PNS) are remote effects of cancer and often are associated with high concentrations of so-called well-characterized onconeural antibodies (anti-Hu, Yo, Ri, CV2/CRMP5, Ma1, Ma2, and amphiphysin) that help to establish the diagnosis [6]. Notably, some “non-classical” PNS (ON, myelitis) have a clinical presentation similar to NMOSD [6–10]. Conversely, previous studies of cancer-associated NMOSD, comprising mainly case reports, postulated a paraneoplastic etiology [11–17], particularly if the tumor expresses AQP4 [18–22]. However, onconeural antibodies were not systematically investigated in NMOSD.

Regarding a previously suggested paraneoplastic etiology in rare cases, we retrospectively investigated the prevalence of onconeural antibodies and malignancies in NMOSD patients.

## Methods

Consecutive patients were identified by an electronic database search. Based on clinical records, NMOSD diagnosis was verified according to recently revised criteria [5]. This approach identified 35 patients with NMOSD who were treated in our clinic (Department of Neurology and Neurophysiology, Medical Center—University of Freiburg, Germany) between 2003 and 2015. Stored serum samples kept at –80 °C from 25 therapy naïve patients were available for analysis. Of these patients, two declined analysis. Finally, 23 patients entered the study. Demographic and clinical data, including anti-AQP4 antibody status, were obtained from patients’ records.

Screening for antibodies targeting intracellular onconeural antigens (Hu, Yo, Ri, CV2/CRMP5, Ma1, Ma2, Zic4, SOX1, Tr, and amphiphysin) was performed on serum samples using a commercial immunoblot with highly purified recombinant antigens according to the manufacturer’s instructions (kindly provided by ravo Diagnostika, Freiburg, Germany).

Dichotomized variables are presented using numbers and percentages; continuous variables are presented using means or medians, range, and standard deviation (SD). The local ethics committee approved the study, and all patients gave written informed consent to the study protocol.

## Results

Table 1 summarizes clinical data of 23 patients fulfilling revised criteria for NMOSD diagnosis and entering the study. Mean age was 44 years (range 19–75, SD 17.2) at disease manifestation, and 49 years (range 20–75, SD 15.8) at diagnosis. Eighteen (78.3%) were female, and 13 (56.5%) were anti-AQP4 antibody positive. Two patients (Table 1: patients #5 and #15) had a malignoma: one had an anaplastic astrocytoma that occurred 7 years after NMOSD manifestation and that progressed to secondary glioblastoma; the other had acute myeloid leukemia (AML) that was treated with stem cell transplantation 4 years before the NMOSD manifestation. Follow-up information was available in all patients with a median duration of 5.0 years (range 0.5–10.0 years, SD 2.7). Remarkably, none had antibodies targeting intracellular onconeural antigens (Hu, Yo, Ri, CV2/CRMP5, Ma1, Ma2, Zic4, SOX1, Tr, and amphiphysin).

**Table 1:** Demographic and clinical characteristics of 23 patients with NMOSD

Patient	Anti-AQP4	Clinical characteristics	Malignoma	Associated autoimmune disease	Duration of follow-up (years)
#1	+	ON and LETM	-	Sjögren’s syndrome	4
#2	-	ON and LETM	-	-	3
#3	-	ON and LETM	-	Hashimoto’s thyroiditis	6
#4	+	LETM	-	-	5,5
#5	-	ON and LETM	Astrocytoma^a^	-	10
#6	+	LETM	-	-	2
#7	-	ON and LETM	-	-	5
#8	-	ON and LETM	-	Hashimoto’s thyroiditis	5
#9	-	ON and LETM	-	-	5
#10	-	ON and LETM	-	Hashimoto’s thyroiditis	9
#11	+	ON and LETM	-	-	0.5
#12	+	LETM	-	Systemic lupus erythematosus	5
#13	+	ON and LETM	-	-	6
#14	-	ON and LETM	-	-	5.5
#15	-	ON and LETM	AML	-	6
#16	+	ON and LETM	-	Sjögren’s syndrome	1
#17	-	ON and LETM	-	-	2
#18	+	ON and LETM	-	-	8
#19	+	ON and LETM	-	Non-differentiated collagenosis	3.5
#20	+	ON and LETM	-	-	8
#21	+	LETM	-	-	0.5
#22	+	LETM	-	Hashimoto's thyroiditis	7
#23	+	ON and LETM	-	-	1

*Abbreviations*: *NMOSD* neuromyelitis optica spectrum disorders, *AQP4* aquaporin-4, *ON* optic neuritis, *LETM* longitudinal extensive transverse myelitis, *AML* acute myeloid leukemia

^a^Anaplastic astrocytoma that progressed to secondary glioblastoma

## Discussion

Inspired by previous reports suggesting a paraneoplastic etiology in rare cases of cancer-associated NMOSD [11–22], this is the first study systematically investigating the seroprevalence of onconeural antibodies (anti-Hu, Yo, Ri, CV2/CRMP5, Ma1, Ma2, Zic4, SOX1, Tr, and amphiphysin) in NMOSD patients.

The principal finding was that all 23 patients’ samples were antibody-negative. However, we acknowledge that the absence of onconeural antibodies does not exclude PNS [6]. In addition, only two patients in our study had a malignancy; yet neoplastic entities (astrocytic brain tumor and AML) are not typically associated with PNS [6]. By contrast, previous reports on putative paraneoplastic NMOSD described associated malignancies that typically occur in PNS patients, predominantly lung and breast cancer [11–22]. Unfortunately, these reports did not systematically investigate onconeural antibodies for comparison with our data. In this regard, there is currently only one case report describing anti-Hu antibodies in a patient with anti-AQP4 positive NMOSD and recurrent thymoma [23].

Limitations of our study were the retrospective design and therefore patients were not systematically screened for occult malignomas. Furthermore, the case number was limited, since serum was available for only 25 of 35 patients (71.4%) previously identified by an electronic database search for those with an NMOSD diagnosis.

## Conclusions

According to our data, the routine screening for onconeural antibodies in NMOSD patients is not mandatory. However, clinicians should pay particular attention in anti-AQP4 negative patients, in patients with a known malignancy or cancer risk factors (e.g. smoking), and/or if clinical presentation is atypical, since paraneoplastic myelitis and/or ON in association with anti-CV2/CRMP5, –Hu or –amphiphysin antibodies might clinically mimic NMOSD [7–10]. Finally, the absence of onconeural antibodies in a typically non-paraneoplastic disorder corresponds to their high specificity for PNS [6]. Finally, larger retrospective trials are necessary to verify these results and to determine the proportion of anti-AQP4 negative NMOSD patients with onconeural antibodies.

## Abbreviations

AML: Acute myeloid leukemia
AQP4: Aquaporin-4
CNS: Central nervous system
LETM: Longitudinally extensive transverse myelitis
NMO: Neuromyelitis optica
NMOSD: Neuromyelitis optica spectrum disorders
ON: Optic neuritis
PNS: Paraneoplastic neurological syndromes
SD: Standard deviation

## References

[1] Wingerchuk DM, Lennon VA, Pittock SJ, Lucchinetti CF, Weinshenker BG (2006). Revised diagnostic criteria for neuromyelitis optica. Neurology.

[2] Wingerchuk DM, Hogancamp WF, O’Brien PC, Weinshenker BG (1999). The clinical course of neuromyelitis optica (Devic’s syndrome). Neurology.

[3] Paul F, Jarius S, Aktas O (2007). Antibody to aquaporin 4 in the diagnosis of neuromyelitis optica. PLoS One.

[4] Jarius S, Wildemann B, Paul F (2014). Neuromyelitis optica: clinical features, immunopathogenesis and treatment. Clin Exp Immunol.

[5] Wingerchuk DM, Banwell B, Bennett JL (2015). International consensus diagnostic criteria for neuromyelitis optica spectrum disorders. Neurology.

[6] Graus F, Delattre J, Antoine J (2004). Recommended diagnostic criteria for paraneoplastic neurological syndromes. J Neurol Neurosurg Psychiatry.

[7] Cross SA, Salomao DR, Parisi JE (2003). Paraneoplastic autoimmune optic neuritis with retinitis defined by CRMP-5-IgG. Ann Neurol.

[8] Ducray F, Roos-Weil R, Garcia P (2007). Devic’s syndrome-like phenotype associated with thymoma and anti-CV2/CRMP5 antibodies. J Neurol Neurosurg Psychiatry.

[9] Jarius S, Wandinger KP, Borowski K, Stoecker W, Wildemann B (2012). Antibodies to CV2/CRMP5 in neuromyelitis optica-like disease: case report and review of the literature. Clin Neurol Neurosurg.

[10] Keegan BM, Pittock SJ, Lennon VA (2008). Autoimmune myelopathy associated with collapsin response-mediator protein-5 immunoglobulin G. Ann Neurol.

[11] Al-Harbi T, Al-Sarawi A, Binfalah M, Dermime S (2014). Paraneoplastic neuromyelitis optica spectrum disorder associated with stomach carcinoid tumor. Hematol Oncol Stem Cell Ther.

[12] Cai G, He D, Chu L, Dai Q, Xu Z, Zhang Y (2016). Paraneoplastic neuromyelitis optica spectrum disorders: three new cases and a review of the literature. Int J Neurosci.

[13] De Santis G, Caniatti L, De Vito A, De Gennaro R, Granieri E, Tola MR (2009). A possible paraneoplastic neuromyelitis optica associated with lung cancer. Neurol Sci.

[14] Moussawi K, Lin DJ, Matiello M, Chew S, Morganstern D, Vaitkevicius H (2016). Brainstem and limbic encephalitis with paraneoplastic neuromyelitis optica. J Clin Neurosci.

[15] Mueller S, Dubal DB, Josephson SA (2008). A case of paraneoplastic myelopathy associated with the neuromyelitis optica antibody. Nat Clin Pract Neurol.

[16] Ontaneda D, Fox R (2014). Is neuromyelitis optica with advanced age of onset a paraneoplastic disorder?. Int J Neurosci.

[17] Pittock SJ, Lennon VA (2008). Aquaporin-4 autoantibodies in a paraneoplastic context. Arch Neurol.

[18] Armagan H, Tüzün E, Icöz O (2012). Long extensive transverse myelitis associated with aquaporin-4 antibody and breast cancer: favorable response to cancer treatment. J Spinal Cord Med.

[19] Figueroa M, Guo Y, Tselis A (2014). Paraneoplastic neuromyelitis optica spectrum disorder associated with metastatic carcinoid expressing aquaporin-4. JAMA Neurol.

[20] Frasquet M, Bataller L, Torres-Vega E (2013). Longitudinally extensive transverse myelitis with AQP4 antibodies revealing ovarian teratoma. J Neuroimmunol.

[21] Iorio R, Rindi G, Erra C, Damato V, Ferilli M, Sabatelli M (2015). Neuromyelitis optica spectrum disorder as a paraneoplastic manifestation of lung adenocarcinoma expressing aquaporin-4. Mult Scler.

[22] Verschuur CVM, van der Kooi AJ, Troost D (2015). Anti-aquaporin 4 related paraneoplastic neuromyelitis optica in the presence of adenocarcinoma of the lung. Clin Neuropathol.

[23] Yang HK, Woo SJ, Park W, Hwang J (2014). Paraneoplastic neuromyelitis optica associated with ANNA-1 antibodies in invasive thymoma. BMC Ophthalmol.

